# Psychological predictors of functioning in patients with asthma

**DOI:** 10.3389/fpubh.2026.1841610

**Published:** 2026-06-11

**Authors:** Milena Wojciechowska, Katarzyna Napiórkowska-Baran, Marta Pilaczyńska-Cemel, Agata Kalicka, Grzegorz Przybylski

**Affiliations:** 1 Department of Law and Health Policy, Ludwik Rydygier Collegium Medicum in Bydgoszcz, University of Nicolaus Copernicus in Toruń, Bydgoszcz, Poland; 2 Department of Allergology, Clinical Immunology and Internal Medicine, Ludwik Rydygier Collegium Medicum in Bydgoszcz, University of Nicolaus Copernicus in Toruń, Bydgoszcz, Poland; 3 Department of Pulmonary Diseases, Oncology and Tuberculosis, Ludwik Rydygier Collegium Medicum in Bydgoszcz, University of Nicolaus Copernicus in Toruń, Bydgoszcz, Poland

**Keywords:** asthma, chronic disease, illness acceptance, psychological predictors, psychological traits, self-esteem

## Abstract

**Background:**

Psychological characteristics significantly modify the impact of asthma on overall functioning. The emotional burden associated with the disease interacts with somatic symptoms, while individual differences in stress perception, self-esteem, and coping resources influence patients' adaptation to chronic illness. Therefore, systematic assessment of psychological factors is essential for planning therapeutic interventions and optimizing patient outcomes.

**Objectives:**

The aim of this study was to examine the role of psychological traits in the functioning of patients with asthma and to identify its main predictors.

**Methods:**

The study included patients with asthma and a control group of healthy individuals. Participants were assessed using the Functioning in Chronic Illness Scale (FCIS), the Asthma Control Test (ACT), and standardized psychological instruments measuring variables such as self-esteem, illness acceptance, and depression. Correlation analyses and multiple linear regression models were performed to identify independent predictors of functioning.

**Results:**

Overall functioning in the asthma group was moderate. Regression analyses indicated that psychological variables were significant predictors of functioning outcomes. Illness acceptance and self-esteem emerged as key independent predictors, particularly in the emotional and social domains. Lower psychological resources and higher levels of depressive symptoms were associated with poorer functioning. Although no significant differences in general psychological measures were observed between the asthma and control groups, these variables significantly predicted functioning within the asthma group. A tendency toward poorer functioning was also observed among patients with lower levels of asthma control.

**Conclusions:**

Psychological characteristics constitute important predictors of functioning in asthma. Their systematic assessment may improve identification of patients at risk of impaired functioning and support more targeted therapeutic interventions.

## Introduction

Asthma is a chronic inflammatory disease of the airways, characterized by variable bronchial obstruction and bronchial hyperresponsiveness. It is estimated that asthma affects approximately 3.3%−3.4% of the general population. Symptoms of asthma are observed in 9.1% of children, 11% of adolescents and 6.6% of adults (1). Typical clinical symptoms of asthma include wheezing, shortness of breath, chronic cough and a feeling of chest tightness. The severity of symptoms varies over time and depends on many factors including irritants, allergens, weather changes and respiratory tract infections (2).

Due to its chronic and recurrent course asthma has a significant impact on the multidimensional functioning of patients affecting their physical, emotional, occupational and social domains. The physical symptoms of asthma may lead to reduced tolerance to physical exertion, sleep disturbances and a decline in overall physical performance (2). The disease contributes to the development of anxiety, a sense of threat and a reduced sense of control over one's own health. In some patients depressive symptoms increased stress levels and lowered self-esteem are observed. In the occupational and educational spheres asthma may result in limitations related to career choice, increased school and work absenteeism, reduced productivity and impaired concentration. The disease also contributes to limitations in social and recreational activities, an increased risk of social isolation, stigmatization and dependence on others (3).

The psychological profile of the patient significantly modifies the impact of asthma on quality of life and overall functioning. Research indicates interactions between the emotional burden resulting from the disease and somatic symptoms. Individual differences in stress perception, self-esteem and available coping resources influence the patient's response to chronic illness and its treatment, including adherence to therapeutic recommendations. Taking into account the assessment of a patient's psychological characteristics is important in planning therapeutic interventions and improving the quality of life of patients with asthma (4).

Although previous research highlights the importance of psychological factors in asthma, the evidence remains fragmented and largely based on general measures of psychological functioning. Importantly, there is a lack of studies that specifically apply the Functioning in Chronic Illness Scale (FCIS) in asthma populations. This represents a significant gap, as FCIS provides a multidimensional assessment of functioning in chronic illness that may offer a more comprehensive understanding of patients' psychological and social adaptation (5). Therefore, examining functioning using FCIS in individuals with asthma may contribute to a more nuanced understanding of how psychological profiles are related to everyday functioning in this group.

The aim of this study was to examine the role of psychological traits in the functioning of patients with asthma and to identify its main predictors within the FCIS approach.

## Material and methods

### Participants

A cross-sectional design was used in this study. The study group consisted of 84 patients with asthma aged 38–81 years (mean age: 57.2 years), including 64 women (76.2%) and 20 men (23.8%). Participants were recruited during routine follow-up visits of patients with a confirmed diagnosis of asthma at the Clinic of Allergology, Clinical Immunology and Internal Medicine of University Hospital no. 2 in Bydgoszcz and at the Clinic of Pulmonary Diseases, Allergology and Pulmonary Oncology of the Kuyavian-Pomeranian Center of Pulmonology in Bydgoszcz. All patients who provided informed consent were invited to participate in the study.

The control group consisted of 38 healthy individuals aged 32–81 years (mean age: 60.3 years), including 24 women (63.2%) and 14 men (37.8%). Participants were recruited from the general population. Inclusion criteria included the absence of chronic respiratory diseases, severe mental disorders, and other serious somatic conditions, based on self-report. All participants in the control group provided informed consent prior to participation in the study.

Ethical approval was obtained from the Bioethics Committee (No. 466/2024).

### Tools

The Asthma Control Test (ACT) was used to evaluate asthma control. The Functioning in Chronic Illness Scale (FCIS) was employed to assess the functioning of patients with asthma. Standardized psychological tests were administered to evaluate psychological factors. An overview of the instruments used in the study is presented in Table 1.

**Table 1:** Characteristics of the tests used in the study.

Test	Assessed construct	Number of items	Score range	Interpretation (higher scores)
ACT	Asthma control (last 4 weeks)	5	5–25	Better asthma control
FCIS	Functioning in chronic illness (physical & psychological)	24 (3 × 8)	Scale-specific^*^	Better functioning
AIS	Illness acceptance	8	8–40	Higher illness acceptance
GSES	General self-efficacy	10	10–40	Higher self-efficacy
MHLC	Health locus of control (internal, powerful others, chance)	18 (3 × 6)	6–36 (per subscale)	Stronger belief in given locus
SES	Global self-esteem	10	10–40	Higher self-esteem
SWLS	Life satisfaction	5	5–35	Higher life satisfaction
BDI	Depressive symptom severity	21	0–63	Higher symptom severity
CECS	Emotional suppression (anger, depression, anxiety)	21 (3 × 7)	21–84	Higher emotional suppression

ACT, Asthma Control Test; FCIS, Functioning in the Chronic Illness Scale; AIS, Acceptance of Illness Scale; GSES, Generalized Self-Efficacy Scale; MHLC, Multidimensional Health Locus of Control Scale assesses health locus of control across three dimensions—internal, powerful others and chance; SES, Rosenberg Self-Esteem Scale; SWLS, Satisfaction with Life Scale; BDI, Beck Depression Inventory; CECS, Courtauld Emotional Control Scale assesses emotional suppression across three domains—anger, depression and anxiety. Higher scores indicate higher levels of the assessed construct unless otherwise specified. ^*^The questionnaire comprises three parts (subscales) measuring: (1) the impact of the illness on the patient, understood as the subjective assessment of the disease's influence on various areas of life; (2) the patient's influence on the course of the illness, referring to beliefs about the ability to control and modify the disease course; and (3) the impact of the illness on the patient's attitudes, reflecting their outlook on the current life situation. Each subscale consists of eight items. The total score is calculated as the sum of points obtained across all subscales. Higher scores indicate better functioning in the context of chronic illness, whereas lower scores reflect a reduced level of functioning. Both the total score and the subscale scores are interpreted within three categories: low, moderate and high (5).

### Statistical analysis

The analyses were conducted using the Python programming language (version 3.13.1) and R (version 4.4.2). Best subset variable selection was performed in R using the *olsrr* package (version 0.6.1). The following packages were used in Python: *pandas* (version 2.3.3)—data processing and descriptive statistics, *seaborn* (version 0.13.2)—data visualization, *plotly* (version 6.3.1)—data visualization and *scipy* (version 1.16.2)—statistical tests.

For the analysis of relationships between variables, Spearman's rank correlation coefficient (ρ) was used as a nonparametric measure of the strength and direction of monotonic relationships. To compare values between two independent groups, either Welch's *t*-test (a version of Student's *t*-test that does not assume equal variances) or the nonparametric Mann–Whitney U test was applied, depending on the distribution of the data. Normality in each group was assessed using the Shapiro–Wilk test.

Four multivariate linear regression models were built, one for each of the four dependent variables: FCIS 1, FCIS 2, FCIS 3, and FCIS. The linear regression model had the form:


$$\begin{array}{c} y_{i}=\beta_{0}+\beta_{1}x_{i}^{\left(1\right)}+\beta_{2}x_{i}^{\left(2\right)}+\ldots +\beta_{p}x_{i}^{\left(p\right)}+\varepsilon_{i},\;i=1,2,\ldots ,n, \end{array}$$

where *y*_*i*_ denotes the value of the dependent variable for the *i*-th observation, $\left(x_{i}^{\left(1\right)},\;x_{i}^{\left(2\right)},\ldots ,x_{i}^{\left(p\right)}\right)$ are the predictors values, β_0_, β_1_, …, β_*p*_ are the model coefficients, ε_*i*_ represents the prediction error, and *n* is the sample size. Predictors included in individual models were selected using the best-subset selection method, which identified the best-fitting sets of explanatory variables for each dependent variable. This method builds 2^*p*^ linear regression models, successively based on all possible subsets of predictors, and returns the best models (with the largest adjusted *R*^2^) with one explanatory variable, two explanatory variables, …, *p* explanatory variables. The adjusted *R*^2^, *AIC*, and *SBC* criteria were then used to select the best of the *p* models. The optimal model for the greatest number of criteria is selected; if this rule is inconclusive, a simpler model (with fewer variables) is selected.

The adjusted *R*^2^ criterion is based on the known coefficient of determination and is defined as


$$\begin{array}{c} adjusted\;R^{2}=1-\left(1-\frac{SSR}{SST}\right)•\frac{n-1}{n-p-1} \end{array}$$

where *SST* and *SSR* are the regression and the total sums of squares, respectively. The adjusted *R*^2^ is expected to be as large as possible.

*AIC* is the Akaike criterion:


$$\begin{array}{c} AIC=n\log\left(SSE/n\right)+2\left(k+1\right)+n\left(1+\log2\pi \right) \end{array}$$

where *SSE* denotes the sum of squared errors, *k* is the number of predictors in the model, and log denotes the natural logarithm. The value of this criterion should be as small as possible. The same is for the value of the Schwarz Bayesian criterion *SBC*:


$$\begin{array}{c} SBC=n\log\left(SSE/n\right)+\left(k+1\right)\log n+n\left(1+\log2\pi \right). \end{array}$$

## Results

The total FCIS score in the study group indicated a moderate level of functioning among patients with asthma. A comparable, moderate level was observed across all FCIS subscales. Asthma has a moderate impact on patients' functioning (subscale 1), patients perceived their influence on the course of the disease as moderate (subscale 2) and the impact of the disease on patients' attitudes was also of a moderate level (subscale 3). The ranges of scores and mean values for the FCIS scale are presented in Table 2.

**Table 2:** Summary statistics of the analyzed variables.

Variable	Mean	SD	Min	Median	Max
FCIS	80.55	11.49	47	81	103
FCIS 1	24.08	6.03	11	24	39
FCIS 2	26.49	4.23	13	27	38
FCIS 3	30.13	4.65	17	30.5	38
CECS	50.73	10.23	27	50	88
CECS-anger	16.70	4.10	7	16	28
CECS-depression	16.96	4.08	8	17	28
CECS-anxiety	16.76	3.66	7	17	25
GSES	29.64	3.93	15	30	40
MHLC-internal	23.45	5.88	9	24	36
MHLC-others	26.7	5.41	6	27.5	38
MHLC-chance	21.92	6.15	10	21	36
SWLS	21.45	5.41	7	21	35
AIS	28.12	7.76	8	29	41
BDI	7.71	9.12	0	5	44
SES	29.67	4.95	15	29	41

**Figure 1:**
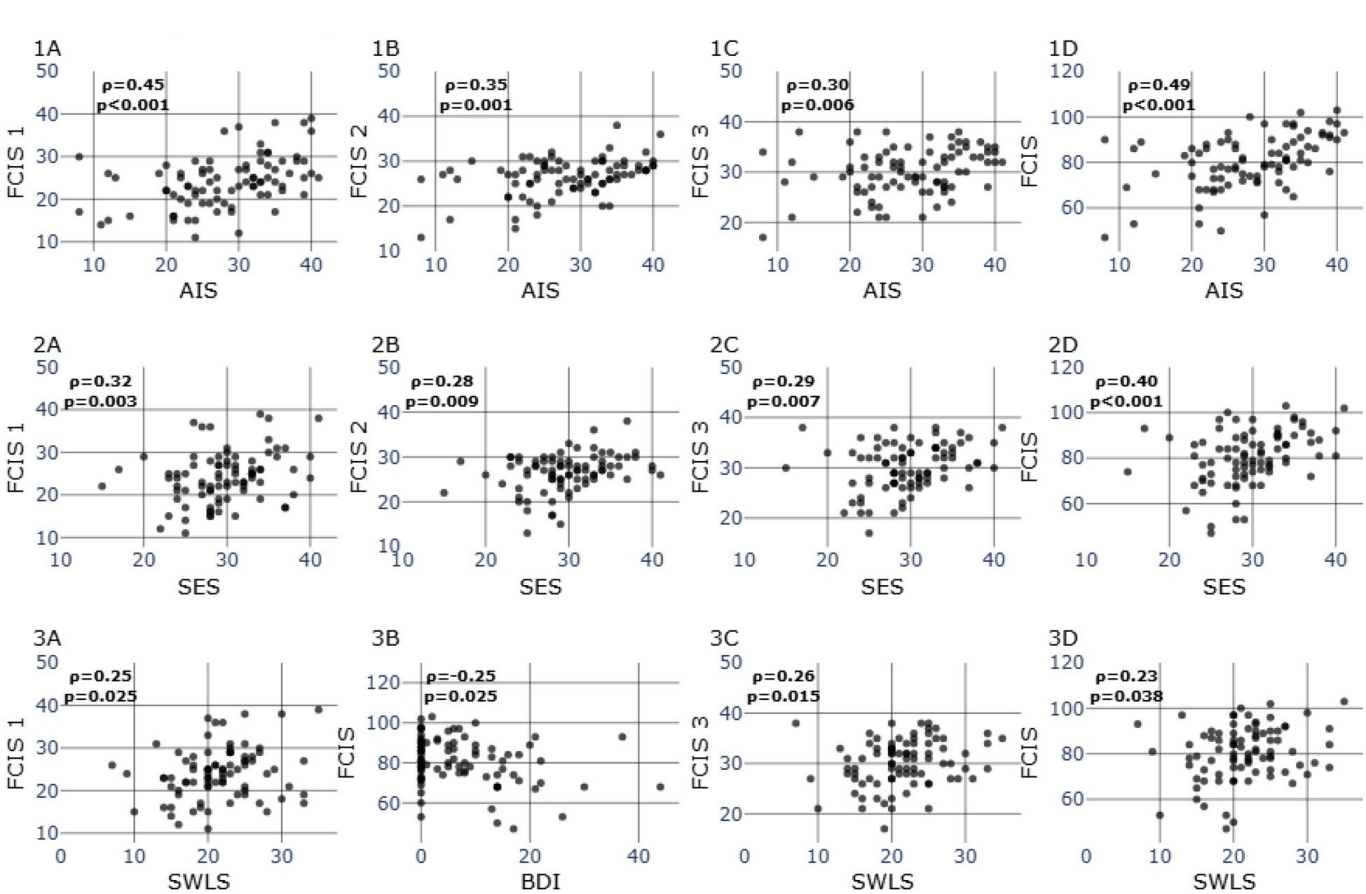
Scatter plots with spearman correlation.

Figure 1 and Table 3 present the results of the Spearman rank correlation analysis, including correlation coefficients and their levels of statistical significance for the study group. For the total FCIS score, statistically significant positive correlations were observed with AIS (1D), SES (2D) and SWLS (3D), in addition to statistically significant negative correlation with BDI (3B). These findings indicate that lower levels of illness acceptance, self-esteem and life satisfaction, in addition to higher severity of mood disturbances are associated with impaired functioning of patients during the course of the disease.

**Table 3:** Spearman's rank correlation results for the study group.

Variable	FCIS_1	FCIS_2	FCIS_3	FCIS	AIS	SWLS	SWLS (control group)
MHLC-internal	ρ = 0.18, *p* = 0.104	ρ = 0.16, *p* = 0.137	ρ = 0.16, *p* = 0.134	ρ = 0.20, *p* = 0.071	ρ = 0.27, *p* = 0.014	ρ = −0.01, *p* = 0.929	ρ =0.20, *p* =0.209
MHLC-others	ρ = 0.09, *p* = 0.418	ρ = −0.10, *p* = 0.371	ρ = 0.10, *p* = 0.388	ρ = 0.03, *p* = 0.814	ρ = −0.08, *p* = 0.473	ρ = 0.00, *p* = 0.966	ρ =0.11, *p* =0.518
MHLC-chance	ρ = −0.08, *p* = 0.485	ρ = −0.14, *p* = 0.210	ρ = −0.15, *p* = 0.161	ρ = −0.18, *p* = 0.104	ρ = −0.16, *p* = 0.150	ρ = −0.24, *p* = 0.029	ρ =0.27, *p* =0.093
CECS-anger	ρ = 0.17, *p* = 0.113	ρ = −0.01, *p* = 0.908	ρ = −0.00, *p* = 0.986	ρ = 0.08, *p* = 0.470	ρ = 0.15, *p* = 0.170	ρ = 0.04, *p* = 0.730	ρ =0.06, *p* =0.699
CECS-depression	ρ = −0.08, *p* = 0.468	ρ = −0.05, *p* = 0.659	ρ = −0.19, *p* = 0.089	ρ = −0.14, *p* = 0.214	ρ = −0.07, *p* = 0.552	ρ = −0.20, *p* = 0.073	ρ =0.07, *p* =0.664
CECS-anxiety	ρ = 0.17, *p* = 0.130	ρ = 0.02, *p* = 0.891	ρ = 0.08, *p* = 0.488	ρ = 0.15, *p* = 0.179	ρ = 0.10, *p* = 0.383	ρ = −0.22, *p* = 0.040	ρ =0.10, *p* =0.558
CECS	ρ = 0.11, *p* = 0.312	ρ = 0.01, *p* = 0.923	ρ = −0.00, *p* = 0.995	ρ = 0.07, *p* = 0.532	ρ = 0.07, *p* = 0.545	ρ = −0.16, *p* = 0.145	ρ =0.02, *p* =0.892
GSES	ρ = 0.13, *p* = 0.254	ρ = 0.14, *p* = 0.200	ρ = 0.18*, p* = 0.101	ρ = 0.20, *p* = 0.072	ρ = 0.18, *p* = 0.108	ρ = 0.16, *p* = 0.158	ρ =0.56, *p* < 0.001
SWLS	ρ = 0.25, *p* = 0.025	ρ = 0.05, *p* = 0.631	ρ = 0.26, *p* = 0.015	ρ = 0.23, *p* = 0.038	ρ = 0.27, *p* = 0.012		
AIS	ρ = 0.45, *p* < 0.001	ρ = 0.35, *p* = 0.001	ρ = 0.30, *p* = 0.006	ρ = 0.49, *p* < 0.001		ρ = 0.27, *p* = 0.012	
BDI	ρ = −0.20, *p* = 0.066	ρ = −0.13, *p* = 0.233	ρ = −0.21, *p* = 0.059	ρ = −0.25, *p* = 0.025	ρ = −0.16, *p* = 0.136	ρ = −0.19, *p* = 0.081	ρ =0.50, *p* =0.001
SES	ρ = 0.32, *p* = 0.003	ρ = 0.28, *p* = 0.009	ρ = 0.29, *p* = 0.007	ρ = 0.40, *p* < 0.001	ρ = 0.29, *p* = 0.007	ρ = 0.46, *p* < 0.001	ρ =0.58, *p* < 0.001

For FCIS 1 and 3 statistically significant positive correlations were found with AIS (1A, 1C), SES (2A, 2C) and SWLS (3A, 3C). This indicates that lower levels of illness acceptance, self-esteem and life satisfaction are associated with a higher subjectively perceived impact of asthma on patients' lives and a more pessimistic attitude toward the future. In addition, for FCIS 2 statistically significant positive correlations were confirmed with AIS (1B) and SES (2B), suggesting that lower levels of illness acceptance and self-esteem are linked to a reduced sense of patients' influence over the course of asthma. Furthermore, statistically significant associations were observed between SWLS and SES, AIS, CECS-Anxiety and MHLC-Others, also between MHLC-Internal and AIS and between AIS and SES (Table 3).

**Figure 2:**
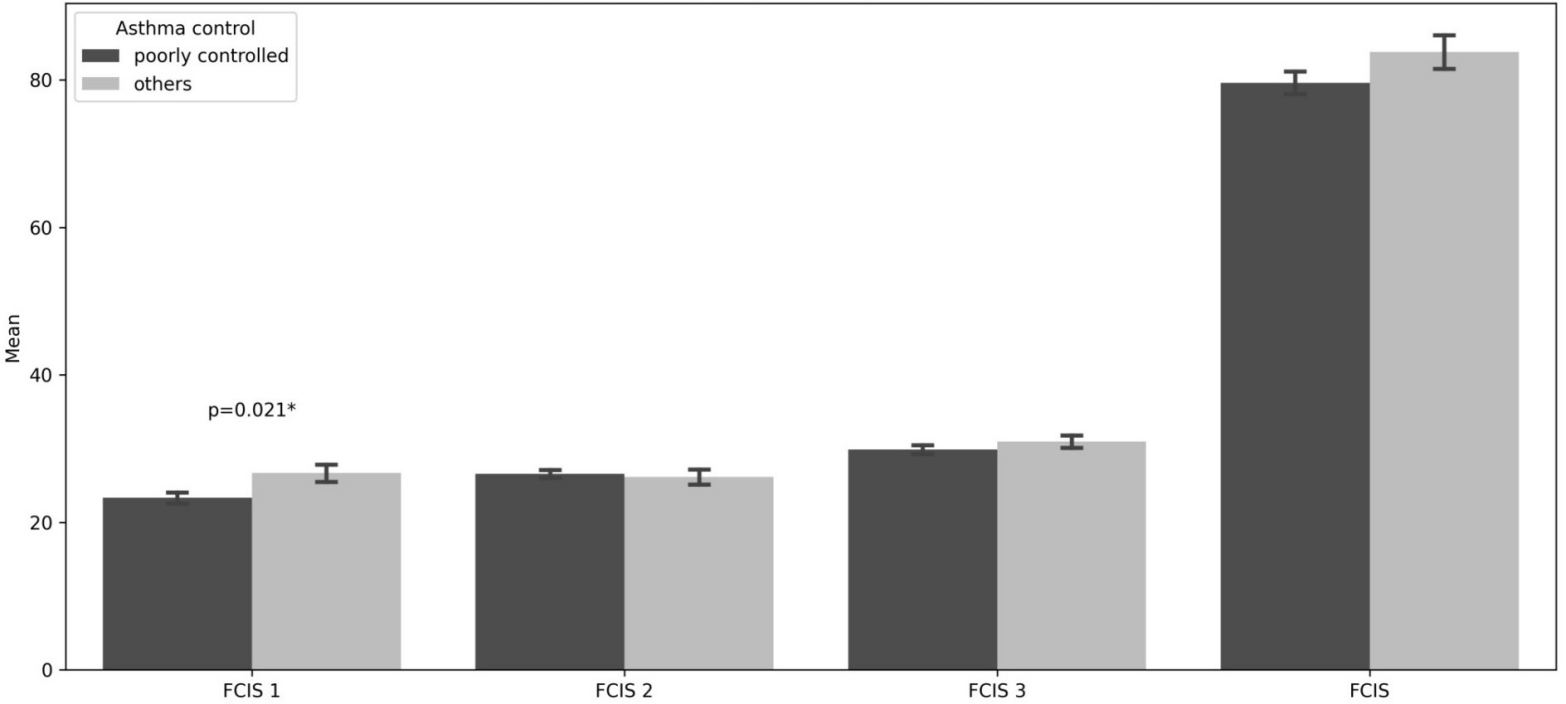
Asthma control groups comparison (Mean ± SE).

Poorly controlled asthma was identified in 65 patients (77.4%), partially controlled asthma in 15 (17.9%), and well-controlled asthma in 4 participants (4.7%). Due to the small size of the well-controlled subgroup, it was combined with the partially controlled group into a single category (“others”). Subsequently, Welch's *t*-test revealed statistically significant differences in FCIS subscale 1 scores between levels of asthma control. As illustrated in Figure 2, these differences were primarily driven by the comparison between patients with poorly controlled asthma and those in the “others” group (*p* = 0.021).

Statistically significant differences between the study group and the control group were observed for all MHLC subscales (Mann–Whitney U test) (Table 4). No statistically significant differences between the groups were found for CECS, SES, BDI, SWLS and GSES scores. In the control group statistically significant correlations were observed between SWLS and BDI, also for SWLS, GSES and SES, indicating that higher severity of mood disturbances and lower self-esteem are associated with reduced life satisfaction in healthy individuals (Table 5).

**Table 4:** Mann–Whitney U test results for the study and control group.

Measure	MHLC-internal	MHLC-others	MHLC-chance	CECS	CECS-anger	CECS-depression	CECS-anxiety	SES	BDI	SWLS	GSES
*p*-value	*p* = 0.043	*P* < 0.001	*p* = 0.001	*p* = 0.896	*p* = 0.155	*p* = 0.793	*p* = 0.762	*p* = 0.841	*p* = 0.098	*p* = 0.437	*p* = 0.260

**Table 5:** Spearman's rank correlation results for the control group.

Variable	CECS_SUMA	CECS_G	CECS_D	CECS_L	BDI	SES	GSES
SWLS	r = −0.04, *p* = 0.813	r = −0.09, *p* = 0.571	r = 0.11, *p* = 0.520	r = −0.15, *p* = 0.363	r = −0.49, *p* = 0.002^*^	r = 0.58, *p* < 0.001^*^	r = 0.56, *p* < 0.001^*^

Linear regression analysis was conducted separately for four dependent variables: FCIS 1, FCIS 2, FCIS 3 and FCIS total (Table 6). Predictors included in the individual models were selected using the best subset selection method, which enabled identification of the best-fitting sets of explanatory variables for each dependent variable. The model fit criteria are presented in Table 7.

**Table 6:** Results of the linear regression models.

Variable	Predictor	Coefficients
FCIS 1	
	Intercept	14.445
	AIS	0.343
FCIS 2	
	Intercept	15.54
	AIS	0.189
	SES	0.191
FCIS 3	
	Intercept	19.872
	AIS	0.139
	SES	0.214
FCIS	
	Intercept	44.024
	AIS	0.625
	SES	0.638

**Table 7:** Model quality metrics—selection of the best model.

Criterion	FCIS 1	FCIS 2	FCIS 3	FCIS
adj. R^2^	(22.76%, 4)	(18.93%, 2)	(12.53%, 5)	(27.69%, 3)
AIC	(524.87, 3)	(468.10, 2)	(491.72, 2)	(633.04, 2)
SBC	(534. 42, 1)	(477.82, 2)	(501.45, 2)	(642.77, 2)

In the FCIS 1 model, AIS emerged as a significant predictor (coeff. = 0.343). This indicates that a one-unit increase in AIS was associated with an average increase of 0.343 units in FCIS 1.

For FCIS 2, the model included two significant independent variables: AIS and SES. AIS was positively associated with FCIS 2 (coeff. = 0.189), indicating that a one-unit increase in AIS corresponded to an average increase of 0.189 units in FCIS 2, holding SES constant. Similarly, SES showed a significant positive association with FCIS 2 (coeff. = 0.191).

A similar pattern of relationships was observed in the FCIS 3 model, in which both AIS (coeff. = 0.139) and SES (coeff. = 0.214) were significant predictors, indicating that higher values of both variables were associated with higher FCIS 3 scores.

In the model for the total FCIS score, AIS and SES were again included as significant positive predictors. A one-unit increase in AIS was associated with an increase of 0.625 units in total FCIS score (coeff. = 0.625), whereas a one-unit increase in SES resulted in an average increase of 0.638 units in FCIS_S (coeff. = 0.638).

The results of the linear regression analyses indicate that the level of illness acceptance was a significant and consistent predictor across all analyzed dimensions of the FCIS scale. In contrast, self-esteem emerged as a significant predictor in the models including FCIS 2, FCIS 3 and the total FCIS score. The direction of the observed associations was consistent across all models, suggesting that higher levels of illness acceptance and higher self-esteem are associated with higher values of functioning indices as measured by the FCIS scale.

## Discussion

The aim of the present study was to analyze the role of selected psychological traits in the functioning of patients with asthma and to identify the most important predictors of this functioning. Correlation analyses made it possible to determine of the relationships between the examined psychological variables and indicators of patient functioning. The use of linear regression models allowed for the simultaneous inclusion of multiple independent variables and the assessment of their significance with regard to specific aspects of patients' functioning.

Functioning in patients with asthma was assessed using the FCIS questionnaire, which encompasses both somatic and psychological dimensions of functioning with a chronic illness (5). In our study, the FCIS score indicates an average level of functioning among patients with asthma. This trend is observed both for overall functioning and across all analyzed subscales. Previous studies shows that the quality of life and functioning of patients with asthma depend on a complex interaction between somatic symptoms, disease control and the patient's psychological resources (6). The degree to which a patient's quality of life is impaired by the severity of specific symptoms or limitations varies and may change depending on variables specific to the individual or potential relationships between them (7). Our study shows that asthma, although it is a chronic and potentially burdensome disease is not always associated with a high level of functional impairment in patients.

Correlation analysis showed that lower levels of illness acceptance, self-esteem and life satisfaction, in addition to higher severity of depressive symptoms were associated with impaired functioning among participants. This pattern of relationships confirms that psychological factors have a significant and multifaceted influence on how patients cope with a chronic illness. Research indicates that psychological factors, such as depression and anxiety are important determinants of quality of life in patients with asthma and may negatively influence both symptom perception and daily functioning. Patients with asthma experience concurrent mental disorders more frequently than the general population (4). Moreover, the relationship between depression and anxiety in individuals with asthma is bidirectional: the disease may lead to increased anxiety about exacerbations and respiratory insufficiency, while anxiety may worsen asthma control (8). Psychosocial factors such as self-esteem and social support have a significant impact on quality of life, as well as on health behaviors and adherence to therapeutic recommendations (9). Previous studies indicate that self-esteem in individuals with asthma is significantly lower than in healthy individuals (10). However, this association was not confirmed in our study. This discrepancy may be related to methodological factors, including the limited sample size and potential differences in demographic characteristics between groups. Nevertheless, it is also possible that certain psychological resources may play a protective role in mitigating the negative impact of the disease. Therefore, these findings should be interpreted with caution, and further research on larger, well-matched samples is needed to clarify this relationship. No significant differences between our study group and the control group in CECS, SES, BDI and GSES scores may indicate that overall levels of self-esteem, emotional control or depressive symptoms do not differ substantially from those observed in the general population. However, their correlational relationships with illness-related functioning reveal their actual impact in the context of asthma.

Our results confirm well-established psychological and behavioral mechanisms in which illness acceptance and self-efficacy play an important role in adaptation to chronic disease. Illness acceptance may facilitate more effective coping with symptoms and better treatment adherence, thereby contributing to improved functioning and quality of life (11). In a cross-sectional study Cai et al. (12), demonstrated that a stronger sense of control and more positive perceptions of one's ability to cope with illness are associated with higher quality of life in individuals with asthma. Moreover, the authors found that negative perceptions of illness consequences and stronger emotional responses are linked to lower quality of life. Significant differences in MHLC scores between our groups suggest, that patients with asthma are more likely than healthy individuals to attribute health control to external factors. Previous studies indicate that individuals with chronic conditions more frequently experience a sense of lack of control over their health status, which may hinder adaptation and effective disease management. A sense of control over one's health and high self-esteem are associated with more active coping strategies, which may mitigate the impact of chronic illness on patients' daily lives (3). Reduced self-esteem is associated with higher levels of experienced stress and an increased negative impact of chronic diseases on patients' daily functioning. Among patients with asthma, lower self-esteem correlates with poorer disease control, whereas higher self-esteem promotes more effective management of symptoms (13).

Our results from analyses comparing functioning according to the level of asthma control suggest a tendency toward impaired functioning in patients with poorly controlled asthma compared with those in the others group. Due to the small size of the well-controlled asthma subgroup, the partially controlled and well-controlled categories were combined. The comparison using Welch's *t*-test indicated a difference between groups (however, this finding should be interpreted with caution, as the limited sample size may have affected the statistical power of the analysis), the direction of the observed changes is consistent with findings from previous studies (14, 15). Research indicates that the level of asthma symptom control is an important factor affecting quality of life and psychoemotional functioning and suboptimal disease control is associated with significantly greater functional limitations and lower subjective quality of life. In up to 75% of asthma patients, a significant relationship is found between quality of life and disease control (7). In a study by Kang et al. (16), significant correlations were demonstrated between the ACT and the EQ-5D quality of life questionnaire. Patients with poorly controlled asthma showed increased impairment across all assessed domains of quality of life, namely mobility, self-care, usual daily activities, pain/discomfort, and anxiety/depression. Although our findings do not allow for definitive causal conclusions, the observable trend, together with other evidence, suggests that asthma control substantially influences patients' psychophysical functioning.

The obtained results clearly indicate that illness acceptance is the strongest and most consistent predictor of all dimensions of functioning assessed by the FCIS. This variable was statistically significant both in relation to individual subscales and the overall score, highlighting its fundamental importance in adapting to a chronic disease such as asthma. Illness acceptance plays a crucial role in maintaining the psychological wellbeing of individuals coping with health problems. It helps reduce emotional tension, alleviates anxiety and depressive symptoms, and facilitates adaptation to the new life circumstances imposed by the disease (17). It has been shown that patients with asthma who demonstrate higher levels of illness acceptance adhere better to medical recommendations, which improves asthma control and overall health status. This, in turn, strengthens their sense of control over their health and enhances life satisfaction (18, 19). Conversely, a low level of illness acceptance may increase stress and hinder the adaptation process, negatively affecting patients' emotional and social functioning (17).

The second significant predictor was self-esteem, which was significantly associated with the FCIS 2 and FCIS 3 subscales as well as the total FCIS score. This result indicates that self-esteem influences the patient's perceived ability to affect the course of the disease, in addition to shaping their attitude toward life circumstances resulting from the illness. The lack of significance of self-esteem in the model for FCIS 1 may suggest that the subjective assessment of the impact of the disease on various areas of a patient's life depends less on a global self-evaluation and more on acceptance of the illness itself. Simultaneously, the positive direction of the associations in the other models indicates that higher self-esteem may promote better emotional and social functioning in patients with asthma. Sidhu et al. (20), demonstrated that patients with asthma exhibit reduced self-esteem and a strong sense of stigmatization, resulting from the chronic course of the disease, limitations in performing daily activities and avoidance of social participation. The authors emphasize the need to provide psychological support to these patients, which may significantly enhance treatment effectiveness.

The significance of both illness acceptance and self-esteem in models including the total FCIS score suggests that the functioning of patients with asthma is multidimensional and shaped by the interplay of various psychological resources. Illness acceptance may serve as a core adaptive mechanism, while self-esteem may enhance patients' ability to maintain a satisfactory level of functioning despite health-related limitations. The consistent direction of the observed associations across all models reinforces the reliability of these findings and indicates the stable nature of the relationships identified.

## Conclusion

The results of the present study indicate that psychological traits are important determinants of functioning in patients with asthma, particularly with regard to the subjective perception of disease-related limitations and overall wellbeing. Taking these factors into account in clinical practice may contribute to more comprehensive care, in which psychological interventions and psychoeducation constitute an important complement to standard medical therapy. Such interventions, aimed at increasing illness acceptance, strengthening self-esteem and improving coping strategies, show considerable potential in contemporary research on the psychosocial aspects of chronic diseases.

## Limitations

This study has some limitations that should be considered when interpreting the results. First, its cross-sectional design precludes conclusions about causal relationships between psychological characteristics and functioning in patients with asthma. Second, the relatively small sample size, particularly in the subgroup of patients with well-controlled asthma, may have reduced the statistical power of comparative analyses and contributed to the lack of statistical significance in *post hoc* tests.

## Data Availability

Data are available from the corresponding author upon reasonable request.
